# Allergological Importance of Invertebrate Glutathione Transferases in Tropical Environments

**DOI:** 10.3389/falgy.2021.695262

**Published:** 2021-06-14

**Authors:** Josefina Zakzuk, Ana Lozano, Luis Caraballo

**Affiliations:** Institute for Immunological Research, University of Cartagena, Cartagena, Colombia

**Keywords:** allergen, house dust mite, Ascaris, cockroach allergen, glutathione S-transferase, IgE, tropics

## Abstract

Glutathione-S transferases (GSTs) are part of a ubiquitous family of dimeric proteins that participate in detoxification reactions. It has been demonstrated that various GSTs induce allergic reactions in humans: those originating from house dust mites (HDM), cockroaches, and helminths being the best characterized. Evaluation of their allergenic activity suggests that they have a clinical impact. GST allergens belong to different classes: mu (Blo t 8, Der p 8, Der f 8, and Tyr p 8), sigma (Bla g 5 and Asc s 13), or delta (Per a 5). Also, IgE-binding molecules belonging to the pi-class have been discovered in helminths, but they are not officially recognized as allergens. In this review, we describe some aspects of the biology of GST, analyze their allergenic activity, and explore the structural aspects and clinical impact of their cross-reactivity.

## Introduction

Glutathione-S transferases (GSTs) are a part of a ubiquitous family of dimeric proteins that have triggered the interest of different biology-related disciplines due to their involvement in drug resistance, carcinogenesis, and allergy. Most of these isoenzymes participate in detoxification reactions, coupling reduced glutathione (GSH) to xenobiotics and/or endogenous substances, such as bilirubin and steroids. They have allergological importance for multiple reasons: several of them are epidemiologically relevant as environmental sensitizers and due to their sequence and structural conservation even among phylogenetically distant species, they may mediate cross-reactivity among invertebrate allergen sources, such as cockroach, house dust mites (HDM), and helminths. Also, probably because of their abundance and immunogenicity, some helminth GSTs have been evaluated in pre-clinical studies and clinical trials as vaccines to prevent helminthiasis (1, 2). Altogether, it means that it is necessary to determine the allergenic activity of these IgE-binding molecules and the clinical impact of their cross-reactivity, including potential allergic reactions after anti-helminth vaccination. Although it is still an unexplored field, immunomodulatory properties have been detected in certain parasite GSTs, of which some of them interfere with type 2 responses or strengthen immunosuppression (3–5). In this review, we describe some aspects of the biology of GST, analyze their allergenic activity, and explore the structural reasons and clinical impact of their cross-reactivity.

## Biological Aspects of GSTs

The main enzymatic reaction of GST consists of the conjugation of the thiol group of GSH to chemical electrophilic centers. The resulting conjugates are less reactive or more water-soluble, facilitating its excretion (6–8). Other less common functions of GST have been described in invertebrates. Meyer et al. purified a sigma-GST from the helminth *Ascaridia galli* with homology to a GSH-dependent prostaglandin-H D-isomerase and high activity and specificity in the GSH-dependent isomerization of prostaglandin H to prostaglandin E, a lipid mediator associated with suppression of host immunity (9). Two kappa-GSTs from *Caenorhabditis elegans*, were located in peroxisomes and mitochondria, and, by RNA interference experiments, were found to be involved in oxygen consumption and lipid metabolism (10). Some GSTs also catalyze the selenium-independent reduction of peroxide-containing compounds (11). Arthropod GSTs may confer resistance *via* direct metabolism, sequestration of chemicals, or metabolizing secondary products. To date, GST activity has been associated with resistance to all main classes of insecticides (12). In helminths, the main detoxifying system is mediated by GSTs; CYP450, another important system found in animals, is not present in most parasites (13). GST content and activity are usually investigated through affinity chromatography and using 1-chloro-2,4-dinitrobenzene (CDNB) substrate, since most of these enzymes may catalyze reactions. However, due to their importance in insecticide and worm chemotherapy resistance, it is common to use high-throughput methods, such as quantitative transcriptomics, functional genomics, and structural studies, for their characterization (12).

### Classification

Members of this family are classified according to their cellular localizations into three major families, i.e., cytosolic, mitochondrial/peroxisomal, and microsomal GSTs (14). Cytosolic GSTs are the most abundant and best characterized. In general, they are 23–28 kDa molecules that are functionally active as dimers. They contain the fundamental signature of an N-terminal domain that binds GSH and an alpha-helix conformation in the C-terminal domain, also known as the substrate-binding site. Among cytosolic GSTs, several classes (mu, alpha, pi, theta, sigma, zeta, and omega) are widely distributed in nature. Others are more specific to certain kingdoms or phyla. Epsilon GSTs, for example, have been identified only in insects and delta GSTs in certain arthropod classes (15). All the known allergens belong to mu, sigma, or delta classes. Also, IgE-binding molecules belonging to the pi-class have been discovered in helminths (16), but have not been officially recognized as allergens by the IUIS. Although most GSTs conserved a general structural pattern, isoforms from different classes present low sequence similarity (14, 17). It is expected that GSTs from different classes are not cross-reactive (18), but this is not a rule; for example, cross-reactivity between the sigma Bla g 5 and a pi-GST from the nematode *Wuchereria bancrofti* was demonstrated (16). Knowledge about the class properties of GSTs may guide the research on its allergenic aspects: (a) confirmation of enzymatic activity of a recombinant GST supports a well-folded product, similar to the natural molecule; (b) it must be considered in the evaluation of intrinsic features of allergens that may promote Th2 responses far more than its IgE-binding properties; (c) the natural abundance of GST isoenzymes according to the species is helpful to evaluate the potential cross-reactivity with other sources and understand its clinical impact. For example, a delta-GST from a cockroach may cross-react with an ortholog in HDM (19), but in this last organism, the isoform may not be abundant (20).

#### Sigma Class

Members of this class have been described in mammalians, chickens, insects, helminths, and mollusks (21). The cockroach allergen Bla g 5 (22) and the nematode GST Asc l 3 (23) belong to this class. Their X-ray crystal structures confirm their similarity with other sigma GSTs (24). The first mammalian sigma-class determined structure was from the rat hematopoietic prostaglandin D synthase with GSH bound (25). Human sigma GST is involved in the biosynthesis of prostaglandin D2 (PGD2) from prostaglandin H2 (PGH2) (21). Also, the *Schistosoma hematobium* antigen has been found to retain this enzymatic activity, and murine models have supported the concept that PGD2 production inhibits host immune response and favors parasite survival (26).

#### Mu Class

Its members have a particular loop between the β2 sheet and the α2 helix, and they typically attach GSH to highly electrophilic compounds (27). All described mite GSTs (Der p 8, Der f 8, Blo t 8, and Tyr p 8) correspond to the mu-class (24). Even though GSTs from this class have been reported in different helminth species, there is no description of a mu-GST allergen in helminths (28, 29). In *Fasciola hepatica*, the common liver fluke, mu-GST expression is highly reactive to chemotherapy, and its activity is involved in protection against xenobiotics, even in a more important way than its sigma member (29). Vaccination with a DNA construct coding for this GST induced humoral response of different isotypes, including IgE but was dominated by the IgG1 isotype (30).

#### Delta Class

Together with epsilon, these GST classes are monophilic for arthropods. Delta is the major cytosolic GST class in insects and their genes are organized in large clusters (31). Allergenic delta-GSTs have been identified in cockroaches (32, 33). Their presence in other arthropods different from insects has not been fully investigated, although there are reports of delta-GSTs in Arachnida, including the Acari subclass (34, 35). Dougall et al. identified a delta-like GST in *Dermatophagoides pteronyssinus*, but this sequence also showed similarity with epsilon-GSTs (20). Classification of GST may be imprecise since certain proteins may have homology to more than one class of GST. In this case, studies of the gene locus, substrate-specificity experiments, and evidence of cross-reactivity with a specific class may help to better define its classification. Insects tend to have numerous delta-GST paralogous genes (34). For example, delta class represents 39% of all GST genes in *A. gambiae* (31). On the contrary, in Acari, mu-GSTs tend to be relatively more abundant (35). Delta-GST activity has been linked to resistance to insecticides and acaricides. Their expression may also be raised by exposure to toxic compounds (36). This has also raised the concern of higher allergenic activity (37) of HDM in cities with poor air quality associated with diesel and other environmental contaminants, as described for Der p 8 (38).

## Allergens

**Figure 1:**
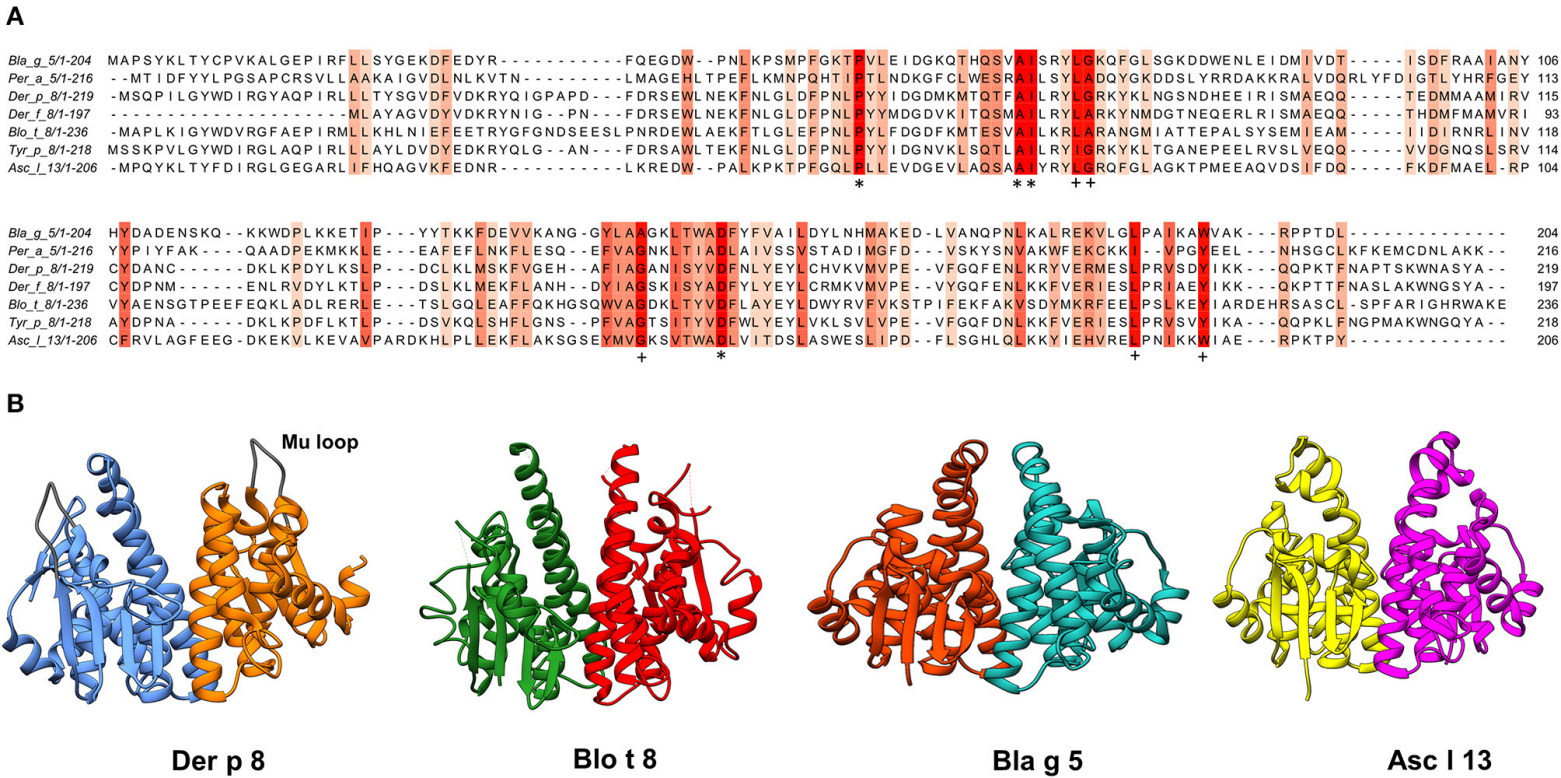
**(A)** Multiple sequence alignments of reported GST Allergens. The most conserved columns have the most intense color, and the least conserved are the palest. A threshold score of 6 was used (conserved amino acids are those with six or more shared properties out of 10) (39). Columns with the same amino acids are marked with “*”, and columns with mutations where all properties are conserved are marked with “+”. AMAS method of multiple sequence alignment analysis was used to calculate the physicochemical properties conserved for each column **(B)** Ribbon diagrams of the structures of allergenic GSTs were generated in UCSF Chimera (40) using data from the PDB database (41), Der p 8 (PDB: 4Q5Q), Blo t 8 (PDB: 4Q5N), Bla g 5 (PDB: 4Q5R) and Asc l 13 (PDB: 4Q5F).

It has been demonstrated that several GSTs induce allergic reactions in humans, those originating from HDM, cockroaches, and helminths being the best characterized (Figure 1). After a systematic search in MEDLINE using the words “glutathione transferase,” “allergen,” and “IgE,” we identified 24 out of 229 references reporting information about IgE-binding properties in 12 GSTs. When searching in Allergome.org, we identified 30 entries with this biochemical function belonging to different sources: cockroaches (32, 33), HDM (37, 42, 43), scabies (20), fungi (44), plants (45, 46), and helminths (23). Only 10 of them are officially recognized as allergens by the WHO/IUIS Allergen Nomenclature Committee and eight are derived from invertebrates. Two GST from helminths (Asc s 13 and Asc l 13) are officially recognized allergens, but in total, eight molecules are listed, of which three are predicted *in silico* as allergenic without any experimental evidence of at least IgE binding. Table 1 shows the most important features of the eight invertebrate GSTs officially reported as allergens, showing that most of them have positive provocation assays, confirming their allergenic activity. Mueller et al. solved the 3D structure of four of these allergenic GSTs and confirmed the global structural similarity among them; however, a relatively low level of conservation of surface-exposed residues was observed (24). Except for Der p 8 and Der f 8, the allergenic activity (47) of most of them have been evaluated by *in vivo* or *in vitro* provocation tests (Table 1), suggesting that they may have a clinical impact. However, their involvement in disease presentation has not been assessed (i.e., case-control studies or avoidance studies). It would be interesting to explore other non–IgE-mediated effects of GST that may predispose to allergic inflammation. Evidence of this was obtained for Der f 8 as explained below (48). Also, immunomodulatory properties influencing type 2 responses have been described for a helminth GST from *Schistosoma* that reduces intestinal inflammation through eosinophil-dependent modulation of pathological Th1 responses (49).

**Table 1:** The allergenic activity of glutathione-S transferase (GST) from invertebrates officially named as allergens.

**Allergen**	**Species**	**Class**	**Provocation test *in vivo***	**Provocation test *in vitro***	**PCA animal model**	**Non-IgE–induced inflammation**
Bla g 5	*Blattella germanica*	Sigma	ST	n.d.	Yes	n.d.
Per a 5.0101	*Periplaneta americana*	Delta	n.d.	BAT	n.d.	n.d.
Per a 5.0102		Delta	n.d.	BAT	n.d.	n.d.
Der p 8	*Dermatophagoides pteronyssinus*	Mu	n.d.	n.d.	n.d.	n.d.
Der f 8	*Dermatophagoides farinae*	Mu	n.d.	n.d.	n.d.	Yes
Tyr p 8	*Tyrophagus putrescentiae*	Mu	n.d.	HR	n.d.	n.d.
Blo t 8	*Blomia tropicalis*	Mu	ST	n.d.	n.d.	n.d.
Asc s 13	*Ascaris suum*	Sigma	ST	n.d.	n.d.	n.d.

*ST, skin prick test; HR, histamine release; BAT, basophil activation test*.

### House Dust Mites

#### Dermatophagoides pteronyssinus

Described by O'Neill et al. (50), Der p 8 was the first reported GST allergen (Der p 8.0101). In this review, an IgE-binding frequency of 40% in 193 *D. pteronyssinus* allergic patients was observed (43). Another isoform was isolated and produced by research group of Dr. YK Chua; interestingly, due to only six amino acid replacements, this isoform had an isoelectric point (pI) of 8.5, which is significantly different from the 6.1 predicted pI of Der p 8.0101. Characterization of the native GST fraction by 2D-gel electrophoresis and western blot analysis (with anti-Der p 8 polyclonal antibodies) indicated that there were at least eight GST isoforms and confirmed the existence of the cloned allergen. Taiwanese patients showed 96 and 84% of IgE reactivity with native and recombinant Der p 8, respectively. Results from Malaysia and Singapore showed lower sensitization rates: 65 and 75%, respectively. Huang et al. also reported moderate cross-reactivity between Der p 8 and the American cockroach native GST isolated by affinity chromatography (42). We also performed data mining of the *D. pteronyssinus* published genome, finding eight entries sharing different degrees of sequence identity with Der p 8.0101 (41–99%) and were annotated as mu-like GSTs. Characterization of their biochemical features by ProtParam indicated that their *pI*s also ranged from five to nine as the results previously observed at the proteomic level (42). It is expected that, as in most arthropods, HDM harbor genes coding for other GST classes. Dougall et al. (20) by a random screening of clones from a cDNA library, found a delta-like GST sequence (Dp7018E11) with only 26% identity to Der p 8.0101. Furthermore, Liu et al. identified an IgE-binding spot compatible with this sequence after 2D separation of the whole *D. pteronyssinus* extract that was also identified in the genome sequence of this mite (51). There are no reports about the evaluation of its allergenic activity, in neither *in vivo* nor *in vitro* tests.

#### D. farina

Der f 8 was reported by An et al. (52). Serology studies indicate IgE-binding frequencies to Der f 8 in the range of 6–41% (53, 54), but there is no information about its allergenic activity, neither *in vivo* nor *in vitro* tests. An interesting data about Der f 8 is its capacity to induce T cell immunoglobulin mucin domain 4 (TIM4) RNA expression in bone marrow-derived dendritic cells at even higher levels than Der f 1 or Der f 2 (48). The interaction of TIM4 with its cognate ligands, TIM1, can promote Th2-cell proliferation, and the blockade of these molecules remarkably dampened Th2 differentiation and allergic reactions (55, 56). Mice sensitized and challenged with Der f 8 developed a strong pulmonary inflammation and increased local and systemic production of Th2 cytokines. Depletion of GST from HDM extracts abrogated the inflammatory potential normally observed with the complete extract and induced regulatory T cells (48).

#### Blomia tropicalis

Blo t 8 is a 23 kDa allergen with two different sequence entries (sharing 99% identity) in UniProt, and two reported IgE-binding frequencies of 10 and 80% in Singapore and Colombia, respectively (57, 58). The tertiary structure of Blo t 8.0101 was experimentally defined by Mueller et al. and matches with a typically mu-GST (24). Blo t 8 can induce positive skin test reactions (23) and also induce positive passive cutaneous anaphylaxis tests (59). Acevedo et al. described in the natural extract of *Ascaris suum*, a 23 kDa band inhibited by the mite extract. Further MALDI-TOF experiments identified this band as a GST. Blo t 8 and Der p 8.0101 have 35.5% identity. The cross-reactivity between Blo t 8, Der p 8, and Asc l 13 has not been experimentally demonstrated. *In silico* analysis of the 3D structures of these molecules suggest that cross-reactivity between them is low (24). In summary, the allergenic activity of Blo t 8 is confirmed, but more information about its importance on allergic sensitization in tropical populations is needed due to the high variations in seroprevalence between Asia and Latin America. There is evidence that helminth infections boost IgE response to cross-reactive environmental allergens, such as HDM and cockroaches (16, 60) and, possibly, the national prevalence of helminthiasis plays a role in this difference on Blo t 8 sensitization rates. In Singapore, a high-income country, ascariasis is infrequent, in contrast to Colombia where this infection occurs in >20% of the population (61, 62).

#### Storage Mites

Tyr p 8 was isolated from *Tyrophagus putrescentiae* by affinity chromatography. It is a 26 kDa protein sharing 83% of amino acid sequence identity with Der p 8. IgE seroprevalence against Tyr p 8 was 45.3% (48/106) among Taiwanese allergic patients who were sensitized to the mite extract. Pre-adsorption of all sera with *D. pteronyssinus* extract reduced the rate of IgE recognition to 18%, which suggests cross-reactivity among GSTs from these sources. There is evidence of basophil histamine release sensitized with sera from Tyr p 8-allergic patients, confirming its allergenic activity (63).

### Cockroach

#### Blatella germanica

Bla g 5, the GST from *B. germanica*, is one of the most common causes of sensitization among cockroach-allergic patients. Arruda et al. first isolated Bla g 5 by affinity chromatography from the natural extract. Serology analysis revealed 67.5% of sensitization among 40 asthmatic patients reactive to this cockroach (22). This group also cloned the sigma GST (Bla g 5.0101), which is experimentally confirmed as a GST and is officially recognized as an allergen. Interestingly, Bla g 5 is the only allergen with a high correlation between specific IgE and wheal size in the skin test (64). Satinover et al. measured using the CAP assay IgE reactivity to different cockroach components, demonstrating in 118 patients that, together with Bla g 2, Bla g 5 dominates IgE responses (65). In summary, this allergen is an epidemiologically relevant component, whose allergenic activity has been confirmed *in vivo* by skin tests.

Jeong et al. isolated a delta-Bla g 5 isoform with just 15% identity with the sigma class and limited cross-reactivity. This isoform showed higher enzymatic activity when CDNB and 1,1,1-trichloro-2,2-bis(p-chlorophenyl) ethane and 4-hydroxynonenal were used as substrates, but its IgE-binding frequency was lower (17.9%) compared to the sigma class (20.5%) in cockroach-sensitized patients from Seoul, Korea. There is no evidence of its allergenic activity (33).

It would be interesting to explore whether there is a relationship between low socioeconomic background and higher sensitization to Bla g 5, as it has been observed for the complete German cockroach extract (66). Possibly, due to lower hygienic conditions, higher environmental levels of *B. germanica* allergens are found in low-income dwellings, as detected with Bla g 2 in the United States (67). Since helminth infections are linked to poverty, it may be hypothesized that underdeveloped countries may have higher sensitization to allergens with cross-reactive homologs present in helminths. Santiago et al. demonstrated that filarial-infected patients had higher IgE levels to Bla g 5 than non-infected donors living in the same endemic regions (16); however, this is not easy to explore because of variations in its geographical distribution. *B. germanica* is one of the most common cockroach species found in Canada, Europe, and the United States (68). Despite being a cosmopolitan species and its origin being tropical Asia (69), it is present but not abundant in the tropics. Meanwhile, in tropical and sub-tropical regions, *Periplaneta americana* is the most prevalent indoor species (70). Barbosa et al. found only 7% of sensitization to sigma Bla g 5.0101 in 53 subjects in Brazil (71).

#### Periplaneta americana

A GST-like sequence from this cockroach was reported in 2006 in GenBank by Chew et al., but without data about its allergenicity. PCR primers based on this sequence led to the identification of the two isoforms with 99% homology by different groups, officially named as Per a 5.0101 and Per a 5.0102. Wei et al. produced the 0.0102 isoform as a recombinant protein in Baculovirus and *E. coli* systems. Chinese *P. americana*-allergic patients showed 25% (4/16) of IgE reactivity to this isoform. Both recombinant versions induced basophil activation as measured by the CD63-based flow cytometry assay (19). Per a 5 was described as a delta-GST with 81% homology to BGGSTD1 and low similarity to sigma GST allergens (15 and 13% to Bla g 5 and Der p 8, respectively). Sookrung et al. isolated Per a 5.0101 from *P. americana* collected in a dwelling in Thailand and characterized its whole repertoire of GST isoenzymes. They found that native and recombinant *P. americana*-GSTs were enzymatically active and 100% of IgE reactivity in sera of all *P. americana*-allergic patients living in Thailand (32). Proteomic analysis indicated that the native GST comprises three isoforms of delta and sigma classes. Interestingly, all isoforms interacted with serum IgE of the cockroach-allergic subjects. IgE reactivity to the original sequence published by Chew et al., (98–99% similar to the other two official isoforms) was 47% in Indian allergic patients. In this same review, it is reported that Per a 5 inhibited IgE binding to cockroach and HDM extracts (72). Per a 5 was also tested in Taiwan with no differences in IgE reactivity between allergic rhinitis (66%) and asthmatic (55%) patients (73). In summary, *P. americana* GST is an allergen with two isoforms with confirmed allergenic activity and epidemiological importance in tropical Asia. Data from Latin America is missing.

### Helminths

Different helminth GSTs (*S. haematobium, S. mansoni*, and *W. bancrofti*) can induce IgE antibodies in infected individuals (16, 74, 75), but only *A. lumbricoides* and *A. suum* GSTs have been reported as allergens. In the tropics, *A. lumbricoides* is of great relevance; first, it causes the most common soil-transmitted helminthiasis (61); and second, several epidemiological surveys have found that ascariasis is a risk factor for asthma and atopy (76). We cannot rule out the importance of other helminths GSTs as allergens since this has not been systematically explored.

#### *Ascaris* spp.

The native *Ascaris lumbricoides* GSTs (nGSTA) purified by Acevedo et al. contained six isoforms that bind IgE (23). To date, the best-studied of these isoforms is the sigma class GST, currently reported as the Asc l 13 allergen by the WHO/IUIS Allergen Nomenclature Sub-Committee. Its sequence is completely identical to Asc s 13. The allergenic activity of the recombinant isoform (rAsc s 13) and nGSTA have been evaluated in asthmatic patients exposed to *A. lumbricoides*. In this population, there was a sensitization frequency of 19.5% in asthmatic patients and 13.2% in controls to rAsc l 13; and the strength of IgE levels to rAsc l 13 among asthmatic patients was significantly higher compared to mite and cockroach GSTs. Additionally, four out of 10 asthmatics had a positive skin test to nGSTA, proving the allergenic activity of this molecule and its possible clinical relevance for some patients (23).

Results from the birth cohort study FRAAT (Risk Factors for Asthma and Atopy in the Tropics) conducted in a deprived community living in Cartagena, Colombia, indicated that ~20% of children were sensitized to rAsc l 13 at a young age (6 months old), increasing to 45% at 3 years of age. A positive IgE response to Ascaris GST was associated with housing features related to poor hygienic conditions and Ascaris infection (77). Also, a de-sensitization pattern to rAsc l 13 was evident with a 25% lower rate at 6 years of age. This process of desensitization may continue to older age, according to the results of Acevedo et al. in asthmatic patients living in the same city. We found that the frequency of IgE response to rAsc l 13 was significantly greater in children (7–13 years) compared to adolescents and adults (14 years and older) (23). In that sense, IgE sensitization to rAsc l 13 may be associated with exposure to Ascaris, which tends to be more frequent during infancy (77). It is still necessary to further characterize the allergenic activity of Ascaris GSTs and their clinical relevance.

To characterize other allergenic isoforms of Ascaris ssp. in our laboratory, we identified the GST genes predicted by the Davis laboratory at the University of Colorado and expressed them as recombinant proteins. We have analyzed the enzymatic activity and humoral response of an omega-class isoform. The recombinant GST omega-class (rGSTO) has dehydroascorbate reductase and thiol transferase activity reported as well for other omega-class GSTs (51). More than 30% of people from a rural area and endemic for *A. lumbricoides* had IgE against GSTO with a similar frequency to rAsc l 13 (78). Despite corroborating that this sequence codes for a biologically active product, in the correction of the *A. suum* genome assembly submitted by the authors in 2018, the current sequence coding for one omega-GST (F1LF49) has only 90.3% of identity to rGSTO.

**Figure 2:**
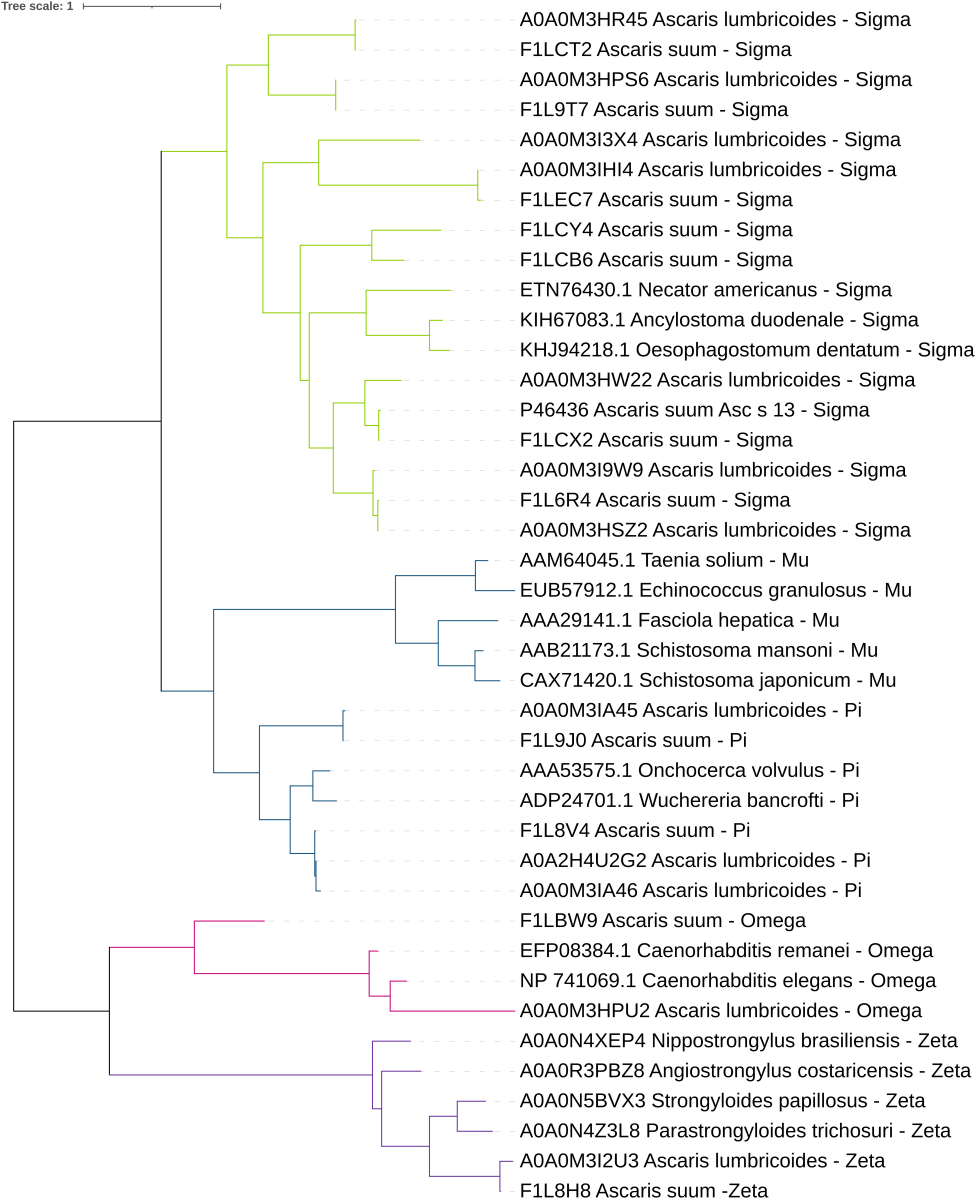
Phylogenetic tree views of cytosolic helminth GSTs. The workflow was created in Phylogeny.fr (81). The amino acid sequences of the identified molecules were aligned using MUSCLE. The phylogenetic tree was constructed using PhyML. The editing was performed in the iTol platform (82).

**Figure 3:**
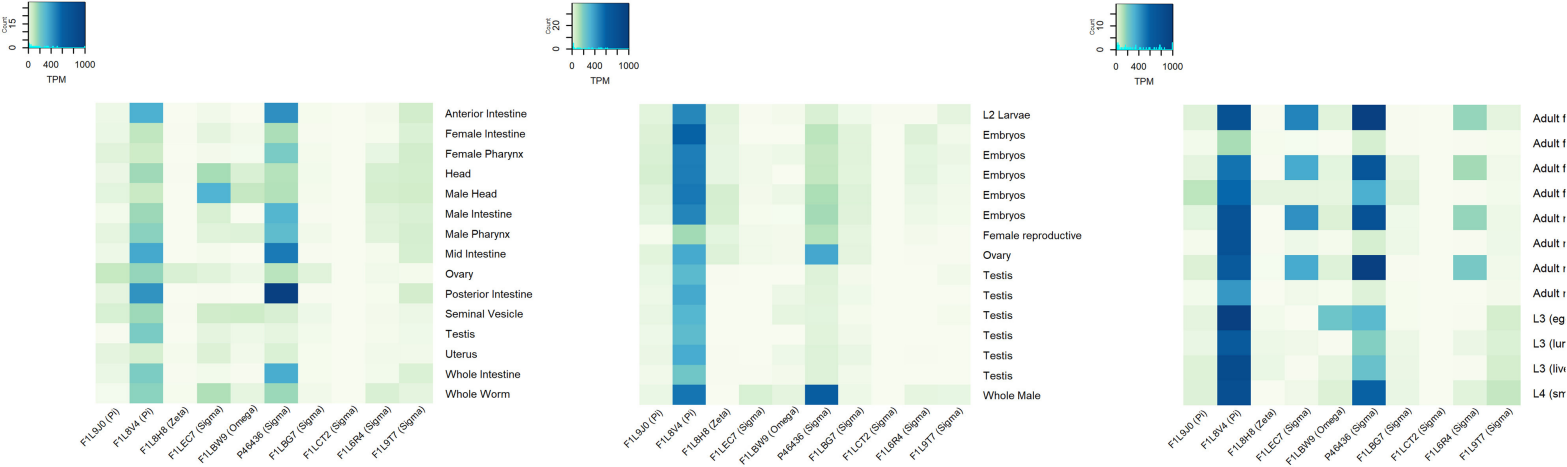
Gene expression profile of GSTs from *Ascaris suum* based on public RNAseq data from experiments SRP013609 **(left)**, SRP005511 **(middle)**, and SRP010159 **(left)**. RNA expression levels in transcripts per million (TPM) in different tissues and lifecycle stages are showed (data from public RNA sequencing studies downloaded from parasite.wormbase.org).

In 2018, the Parasite Genomic group at the Wellcome Trust Sanger Institute made the draft genome assembly and gene predictions of *A. lumbricoides* using a strain from the Republic of Ecuador. Based on this work, we identified 15 GST sequences of *A. lumbricoides* reported in the UniProt database. The sequence and tertiary structure were verified to confirm that these proteins belonged to this protein superfamily. Most predicted products from these sequences had features of the cytosolic subfamily, as the thioredoxin-like fold domain and the GST N-terminal and C-terminal domains. There was only one sequence identified as kappa subfamily GSTs, due to the presence of the DSBA-like thioredoxin domain, and one as microsomal subfamily GST (79). In *A. suum*, according to the genome published by the Davis Laboratory at the University of Colorado in 2018 (80), we identified 14 GSTs in the UniProt database. Most of the proteins have features related to the cytosolic subfamily, two have features of the kappa subfamily and one has features of the microsomal subfamily. Cytosolic GSTs from *A. lumbricoides* and *A. suum* were classified by their homology with different GST classes (Figure 2). At the transcriptional level, we found expression data for 10 out of the 11 cytosolic GSTs (Figure 3). It is generally observed that one pi- and one sigma-isoform (which we also confirmed in the cDNA library) are the most abundant (FILV84 and P46436, respectively). The transcriptional expression also differed between tissues and developmental stages of the parasite. *A. suum* and *A. lumbricoides* are identical at the morphological level, but not in their genomes. DNA sequences coding for GST isoforms in *A. lumbricoides* were in the range of 77.5 and 99.5% identity with another GST from *A. suum*. Only one GST (A0A0M3I3X4) of *A. lumbricoides* did not have a similar sequence in *A. suum* and two sequences in *A. suum* (F1LBW9 and F1LCY4) were not identified in *A. lumbricoides*. The *A. suum* kappa-GST (F1LDD6) had an identity of 68.3% with an *A. lumbricoides* DSBA domain-containing protein with a match length of 182 of 254 amino acids.

## Immunomodulation

Several investigations have pointed out different parasite GSTs as immunomodulatory proteins, including molecules derived from helminths and protists (83). The participation of GST on immunoregulation in mammalian species has also been documented (21). As the focus of this review is invertebrate GSTs, we describe only the evidence on helminth GSTs showing any effect on the immune responses of exposed hosts.

Immunomodulatory properties of sigma-GST (fhGST-si) from *F. hepatica* have been found over mouse BMDCs, reducing their inflammatory response to LPS and inhibiting its capacity to induce Th17 polarization (3). Stimulation of BMDCs with the fhGST-si induced secretion of pro-inflammatory cytokines, such as IL-6, IL-12p40, and MIP2 e IL-10, but caused a partial cellular activation: CD40 upregulation in the absence of CD80, CD86, and MHC-II overexpression. Priming dendritic cells with fhGST-si before the addition of OVA reduced Th17 responses in DO11.10 mice, a transgene strain reactive to an OVA peptide antigen. This same group also tested the recombinant mu-GST from *F. hepatica*, with no observable immunomodulatory effect of this isoform. Aguayo et al. tested the native *F. hepatica* GST (nFhGST), which is majorly comprised of mu-class GST isoforms (96%), in a septic shock mouse model. It was observed that nFhGST significantly suppressed the LPS-induced TNFα and IL1β *in vitro* production by macrophages and the pro-inflammatory cytokine/chemokine storm within C57BL/6 mice elicited by lethal doses of LPS (84).

P28GST, a GST derived from *Schistosoma haematobium* has shown to induce a strong mucosal immune response, associated with IL-10 and Th2 cytokine production in animal models and humans (5, 49). Due to this capacity, it has been tested as a vaccine against schistosomiasis and Crohn's disease. From animal models, it has been observed that administration of Sh28GST is as effective as *S. cercariae* in preventing tissue damage and inflammation (TNF, IL-1β, and IL-6 production) in experimental colitis. It was also found to induce Th2 polarization, characterized by local and systemic production of IL-13 and IL-5. An eosinophilic infiltration in colonic tissue was evidenced in GST-immunized mice, and eosinophil depletion was associated with a loss of the ameliorating effects observed for Sh28GST (49). This molecule was also tested in a curative model, observing therapeutic effects. Administration of P28GST after TNBS-colitis induction decreased gut inflammation, associated with Th1/Th17 response downregulation and the development of alternatively activated macrophages. In this review, the authors also demonstrated that only intact schistosome-derived P28GST was able to induce a significant reduction of the clinical score, in contrast to heat-inactivated P28GST that loses its enzymatic activity (85). Results from a multicenter, open-label, pilot Phase 2a study that evaluated the safety of P28GST administered to patients with mild Crohn's disease (CD) also indicated that this is a safe product and showed a promising effect on reducing disease severity (86).

Data from immunomodulation in humans can be extracted from clinical trials that tested this GST as a vaccine to prevent urinary schistosomiasis. Results from a Phase I study in 24 healthy volunteers who received Sh28GST with alum indicated that immunization with this antigen is mostly safe, with few cases of mild site-infection reactions. In a subset of patients (*n* = 8), whose humoral and cytokine response to Sh28GST-Alum was investigated, it was observed that immunization induced IgG1, IgG2, IgG3, and IgG4 antibodies, in contrast to IgE whose titers did not significantly raise in comparison with baseline. Production of IL-5 and IL-13 was significantly induced after two administrations of rSh28GST in six out of eight vaccinated adults (5). In the Phase III study of this vaccine, 250 children, living in a hyperendemic area of Senegal, were randomized to receive rSh28GST or placebo. The vaccine did not show efficacy to prevent cases of urinary schistosomiasis. The anti-Sh28GST response was characterized by elevated levels of specific IgG1, IgG2, and IgG4 antibodies. In this population, vaccination did increase the specific IgE antibody response with 36.8% positive children in the active group. No cases of anaphylaxis or another systemic reaction after vaccination were reported; however, it is important to highlight that in contexts of natural exposure to helminths, helminths vaccines inducing IgE may induce anaphylaxis, as it was reported for a hookworm vaccine (87).

## Cross-Reactivity

Due to their potential clinical impact, GSTs from environmental sources, such as cockroaches and HDM, are allergens that could be included in component-resolved diagnosis platforms; it will then be necessary to define the clinical impact of cross-reactivity among them. However, there are few studies regarding this topic, probably because the complete repertoire of isoenzymes has not been elucidated in the species of interest. Mueller et al. analyzed *in silico* the potential of IgE cross-reactivity among some of the most studied allergenic GSTs and concluded that the possibility of this phenomenon is low (24). Although Bla g 5 and Asc l 13 are sigma-GSTs, they share scarce surface conservation, not enough to have predicted common epitopes. As expected, less similarity is found among mu- and sigma-GSTs (24). However, results from Santiago et al. suggest that cross-reactivity must be extensively evaluated even when it is not expected. For example, the piGST from *W. bancrofti* is cross-reactive with Bla g 5 despite only sharing 27% of sequence similarity. IgE, IgG, and IgG4 binding to Bla g 5 was inhibited to a significant extent (50–70%) in individual sera collected from cockroach-allergic patients. Modeling and sequence analysis indicated that both molecules may share a lineal epitope in their N-terminal domain (16).

Cockroach allergy has important clinical implications due to its association with emergency room admissions (35, 36). Besides HDM, sensitization to cockroach allergens is the next common risk factor associated with asthma in the tropics (37). In different tropical countries, cockroach sensitization is high, but this is not in agreement with the surprisingly low allergen levels found in house dust (38, 42), and it has been argued that this is because of cross-reactivity. Tropomyosins and GST families are candidates to mediate important clinical cross-reactivity. In a co-exposed population, we have observed a high correlation in IgE levels against helminth, cockroach, and HDM GSTs (23). However, in sera obtained from Bla g 5-positive patients living in the United States, and who are not expected to be exposed to tropical allergenic sources, such as Ascaris and *B. tropicalis*, IgE recognition of Asc l 13 or Blo t 8 was not observed. Moreover, IgE binding to Der p 8 was inhibited only by Der p 8, but not by Bla g 5, Blo t 8, or Asc s 13. These results support the idea that, at least in North America, IgE response to Bla g 5.0101 may reflect genuine sensitization to cockroaches and that cross-reactivity is not relevant. However, in the tropics, a deeper evaluation of cross-reactivity among GSTs might be needed. IgE cross-reactivity is present between Der p 8 and native *P. americana* GST (42) as well as between *B. tropicalis* and *A. suum* native GST (88). Identification of the correct isoforms mediating cross-reactivity is a further step to determine the clinical significance of this phenomenon.

## Discussion

Different GSTs from invertebrates have been identified as molecules with allergenic activity and potential clinical relevance. In addition, some of them are also frequent sensitizers in exposed populations. The identified allergens belong to different GST classes (sigma, mu, and delta); although with a similar global structure, determined by key conserved amino acids and positions, there is low to moderate sequence conservation among them (Figure 1).

Several IgE-binding GSTs have been evaluated for their allergenic activity, being Bla g 5 the best characterized at the recombinant level, but more information on the complete repertoire of GSTs from *B. germanica*, observed in nature, is needed. Defining the allergenic activity will provide better information about the clinical impact of these IgE-binding molecules. In addition, extending the studies about their mechanisms of action for inducing allergic inflammation is necessary to improve our knowledge of allergy pathophysiology and to discover other options of allergy treatment. Their biological activity may be involved in Th2 polarization, which makes it interesting to explore other non-IgE mediated mechanisms that promote allergic responses (48).

Helminth-GSTs can induce different immune responses in the host; while *Schistosoma*-GSTs seem to induce a protective IgE response, the *Ascaris-*GSTs may induce allergic reactions (23). A possible explanation for this difference may be the whole context set up by the infection itself: routes of antigen entry, type 2 adjuvants, and immunosuppressive molecular content (89). There is evidence from animal models that ascariasis could increase the IgE/Th2 responses to bystander antigens (90, 91). In humans, different studies have observed that Th2/IgE hyper-responsiveness induced by Ascaris infection can also boost the IgE responses to HDM allergens, besides its antigens (76, 92). In that sense, there is a possibility that the allergenic response against Ascaris-GST is promoted by other components of *Ascaris*. On the contrary, it is also possible that due to the nature of the infection, Schistosoma spp. has a more immunosuppressive net effect that reduces the chance of potential allergenic molecules, such as GSTs, to elicit IgE-mediated reactions leading to a topic manifestations. Thus far, most of the studies tend to support that schistosome infection reduces skin test responses and allergic responses (93, 94).

The current data suggest that cross-reactivity is not frequent among GST allergens and those detected seem to be non-clinically relevant; however, more research is needed to understand how molecules with a low degree of sequence homology exhibit cross-reactivity. It is expected that GSTs from different classes are not cross-reactive, but an important exception has been described (16). Since living species usually contain genes coding for more than one GST class, it is necessary to evaluate the complete repertoire of GST isoenzymes if cross-reactivity aims to be assessed. It is also important to elucidate the clinical significance of IgE-binding GSTs from helminths due to the risk of allergic reactions to vaccines or immunotherapy strategies that use GST as antigens.

## Author Contributions

JZ and LC: conceived this manuscript. AL and JZ: performed bioinformatic analysis and developed figures. All authors contributed to manuscript writing, reviewing, and editing.

## Conflict of Interest

The authors declare that the research was conducted in the absence of any commercial or financial relationships that could be construed as a potential conflict of interest.
